# Cognitive-Behavioral Therapy for Obsessive–Compulsive Disorder with and without Autism Spectrum Disorder: Gray Matter Differences Associated with Poor Outcome

**DOI:** 10.3389/fpsyt.2017.00143

**Published:** 2017-08-15

**Authors:** Aki Tsuchiyagaito, Yoshiyuki Hirano, Kenichi Asano, Fumiyo Oshima, Sawako Nagaoka, Yoshitake Takebayashi, Koji Matsumoto, Yoshitada Masuda, Masaomi Iyo, Eiji Shimizu, Akiko Nakagawa

**Affiliations:** ^1^Research Center for Child Mental Development, Chiba University, Chiba, Japan; ^2^United Graduate School of Child Development, Osaka University, Kanazawa University, Hamamatsu University School of Medicine, Chiba University and University of Fukui, Suita, Japan; ^3^Department of Neuropsychiatry, Fukushima Medical University, Fukushima, Japan; ^4^Department of Health Risk Communication, Fukushima Medical University, Fukushima, Japan; ^5^Department of Radiology, Chiba University Hospital, Chiba, Japan; ^6^Department of Psychiatry, Graduate School of Medicine, Chiba University, Chiba, Japan; ^7^Department of Cognitive Behavioral Physiology, Graduate School of Medicine, Chiba University, Chiba, Japan

**Keywords:** obsessive–compulsive disorder, autism spectrum disorder, cognitive behavioral therapy, magnetic resonance imaging, voxel-based morphometry

## Abstract

Cognitive behavioral therapy (CBT) is an effective treatment for obsessive–compulsive disorder (OCD) and is also applicable to patients with both OCD and autism spectrum disorder (ASD). However, previous studies have reported that CBT for patients with both OCD and ASD might be less effective than for patients with OCD alone. In addition, there is no evidence as to why autistic traits might be risk factors. Therefore, we investigated whether comorbidity between ASD and OCD may significantly affect treatment outcome and discovered predictors of CBT outcomes using structural magnetic resonance imaging (MRI) data. A total of 39 patients, who were diagnosed with OCD, were enrolled in this study. Of these, except for 2 dropout cases, 15 patients were diagnosed with ASD, and 22 patients were diagnosed with OCD without ASD. Both groups took CBT for 11–20 sessions. First, to examine the effectiveness of CBT for OCD patients with and without ASD, we compared CBT outcomes between the two groups. Second, to investigate how the structural abnormality profile of the brain at pretreatment influenced CBT outcomes, we performed a structural MRI comparison focusing on the gray matter volume of the whole brain in both patients with only OCD, and those with both OCD and ASD. In order to discover neurostructural predictors of CBT outcomes besides autistic traits, we divided our samples again into two groups of those who did and those who did not remit after CBT, and repeated the analysis taking autistic traits into account. The results showed that OCD patients with ASD responded significantly less well to CBT. The OCD patients with ASD had much less gray matter volume in the left occipital lobe than OCD patients without ASD. The non-remission group had a significantly smaller volume of gray matter in the left dorsolateral prefrontal cortex (DLPFC) compared with the remission group, after having partialed out autistic traits. These results indicate that the abnormalities in DLPFC negatively affect the CBT outcome, regardless of the severity of the autistic traits.

## Introduction

Obsessive–compulsive disorder (OCD) is characterized by compulsive behavior, obsessive thoughts, or a combination of both (1). Autism spectrum disorder (ASD), on the other hand, is characterized by restricted, repetitive, and stereotyped patterns of behavior, as well as impairment in social interaction and communication. OCD and ASD can occur simultaneously. A high prevalence of autistic traits has been found in OCD samples both in adults (2, 3) and in children (4). Clinical features of obsessive–compulsive symptoms may be similar between OCD patients with and without ASD. However, the mechanisms of the symptoms seem to be different. OCD patients experience unwanted and intrusive thoughts that, for them, are difficult to cast out of their minds. These thoughts, known as obsessions, often compel them to repeatedly perform ritualistic behaviors and routines, or compulsions, to try to ease their anxiety. On the other hand, individuals with ASD tend not to show distress associated with their cognitive inflexibilities and do not perform rituals to ease their anxiety.

Cognitive behavioral therapy (CBT) is an effective treatment for OCD, and many controlled trials and meta-analyses supported the fact that CBT is effective for OCD [e.g., Ref. (5)]. Although its effectiveness has been demonstrated, some individuals have treatment-resistant symptoms, and they are not able to benefit from CBT. Given this fact, researchers have tried to identify factors that predict the outcome. Comorbidity with ASD, for example, might negatively affect the CBT outcome. CBT for OCD patients with ASD has been less effective than for OCD patients without ASD (6, 7). Bejerot et al. (8) reported that inflexibility, poor social skills, or a strong need for the “just right” feeling are autistic traits in patients with OCD. Therefore, ASD traits might be risk factors leading to poor treatment outcome. However, there is no evidence for why autistic traits might be risk factors. Since identifying the neurostructural markers that might affect and improve the CBT outcome is important, we attempted to examine the effectiveness of CBT for OCD patients with and without ASD, using structural magnetic resonance imaging (MRI) as a means of identifying pretreatment brain markers.

In the past few decades, the most widely accepted model of OCD has focused on brain abnormalities in the “affective” orbitofronto-striatal circuit, mainly consisting of volume reduction in the orbitofrontal cortex (OFC), anterior cingulate cortex (ACC), temporolimbic cortices, thalamus, and striatum (9–18). The circuit including OFC and limbic systems plays important roles in the emotional aspects of behavior (e.g., evaluation and modulation of emotions) and is, therefore, called “affective” circuit (11, 13). More recent neuroimaging studies, however, reveal the involvement of several regions outside the “affective” orbitofronto-striatal circuit. These regions include the dorsolateral prefronto-striatal “executive” circuit, such as the dorsolateral prefronto cortex (DLPFC), parietal, temporal, and occipital lobes (11, 13, 19, 20). The dorsolateral prefronto-striatal circuit is involved in executive functions including planning, decision-making, and shifting from one behavior to another (21), which is therefore called “spatial/attentional” circuit (11) or “executive” circuit (13). In addition, abnormalities of white matter tracts connecting cortical and subcortical nodes of the abovementioned circuits have also been observed in OCD patients (22). White matter alterations in OCD have been found in regions included in the traditional orbitofronto-striatal circuit (19, 23–26), as well as in areas outside this loop such as prefrontal, temporal, parietal, and occipital regions (27–32). These results suggest that not only anatomical brain volumes of gray matter but also anatomical connectivity of white matter are altered in OCD, and these alterations involve a more widespread network of cerebral dysfunctions than previously thought (11, 13). Specifically locating abnormal anatomical circuits including “affective” and “effective” circuits will allow researchers and clinicians to choose more effective treatment options.

Although the results from many studies are inconsistent, brain predictors of OCD have been found in OFC and ACC, which contribute to the “affective” orbitofronto-striatal circuit. For example, in functional neuroimaging studies, there is evidence that pretreatment lower activity in OFC and ACC predicted a better outcome with pharmacotherapy, while pretreatment higher activity in the same regions predicted a better outcome with psychotherapy (33). Using structural neuroimaging, Hoexter et al. (34) found that less gray matter in the ventrolateral prefrontal cortex predicted a better outcome with pharmacotherapy, while more gray matter in ACC predicted a better outcome with psychotherapy. These results showed that the orbitofronto-striatal circuit might be associated with treatment outcome, notwithstanding a difference between treatment modalities (i.e., pharmacotherapy vs. psychotherapy). However, many studies so far have used regions of interest (ROI) analyses to focus on particular brain regions especially associated with the orbitofronto-striatal circuit. Although dysfunctions and structural abnormalities in OFC and striatal areas were found to be associated with OCD pathology (9–18), other evidence indicates that there are widespread dysfunctions and structural abnormalities, represented by the dorsolateral prefronto-striatal “executive” circuit, including DLPFC, parietal, temporal, and occipital lobes (11, 13, 19, 20). Therefore, it was hypothesized that, when we apply whole brain analysis instead of ROI analysis, more widespread brain regions would be revealed as contributing to the CBT outcome.

Our aims in this study were twofold. First, to examine the effectiveness of CBT for OCD patients with and without ASD, which is one of the risk factors for resistance to CBT, we compared CBT outcomes between the two groups. Second, to investigate the profile of pretreatment brain structural abnormalities associated with autistic traits, which might be risk factors for CBT outcomes, we performed a structural MRI comparison in OCD patients with and without ASD. In order to discover neurostructural predictors of CBT outcomes besides autistic traits, the analysis was repeated for those who did and did not remit, taking autistic traits into account. The analysis focused on the gray matter volume of the whole brain.

## Materials and Methods

### Recruitment

**Figure 1:**
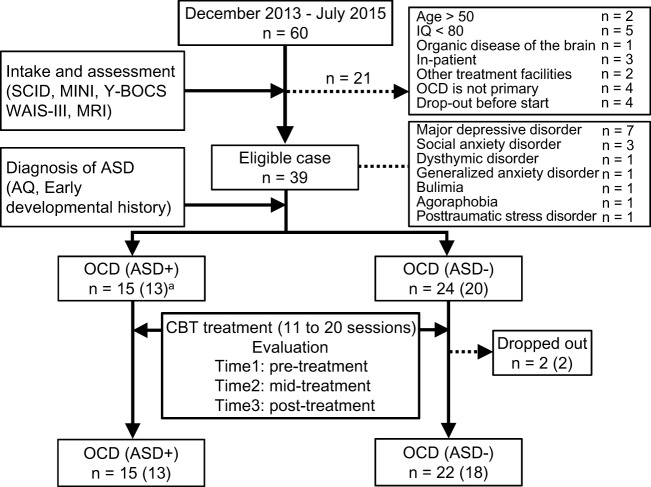
Recruiting process of this study. The number in parentheses means patients who underwent magnetic resonance imaging.

We recruited patients from consecutive referrals to an OCD outpatient clinic at Chiba University Hospital in Japan between December 2013 and July 2015. All patients were diagnosed with OCD by a psychiatrist using the Structured Clinical Interview for DSM-IV Axis I Disorders [SCID-I: (35)]. Figure 1 summarizes participant enrollment and the workflow of this study. Of the 60 patients studied, we included only those patients who satisfied our inclusion criteria: patients aged between 17 and 50, possessing a total intelligence quotient (IQ) over 80, and exhibiting medium to severe obsessive–compulsive symptoms. We assessed their IQ and the severity of obsessive–compulsive symptoms according to the Wechsler Adult Intelligence Scale [WAIS-III: (36)] and the Yale–Brown Obsessive–Compulsive Scale [Y-BOCS: (37)], respectively. For the latter test, patients attaining a total score of 16 or higher were assessed as having medium to severe obsessive–compulsive symptoms (38). Exclusion criteria were the presence of schizophrenia and related disorders, including delusional disorder or psychotic disorders (*N* = 0), substance dependencies (*N* = 0), organic brain diseases (*N* = 1), severe physical diseases (*N* = 0), coexisting Axis I psychiatric disorders that precluded OCD (*N* = 4), medical instability or pregnancy (*N* = 0). Patients unable to attend the weekly treatments were also excluded from the study (*N* = 9). Patients receiving pharmacotherapy were not excluded, but they were requested to keep their pharmacotherapy stable during this study. Thirty-nine OCD patients were eligible, and among these, we defined the comorbid OCD and ASD patients. We obtained detailed developmental histories, life histories, Autism-Spectrum Quotient (AQ) score (39, 40), and profiles of WAIS-III. Then, a psychiatrist (Akiko Nakagawa) and a therapist in charge of each case discussed the case of ASD-suspicious patients, and finally, a psychiatrist (Akiko Nakagawa) decided which ASD-suspicious patients should be diagnosed as comorbid OCD and ASD based on the DSM-IV criteria. Fifteen of the 39 OCD patients were diagnosed with ASD, and the remaining 24 patients were diagnosed as having OCD without ASD. The OCD patients with ASD were designated as the OCD (ASD+) group, and those without ASD were designated as the OCD (ASD−) group. There were two dropouts in the OCD (ASD−) group. Therefore, we analyzed the data of 15 patients in the OCD (ASD+) group and 22 patients in the OCD (ASD−) group.

### Treatment

In this study, we administered CBT for OCD according to a Japanese guidebook (41) designed for adult outpatients with OCD. Fifty minute weekly CBT sessions were scheduled. The content and structure of CBT were as follows: the first session included medical history taking, the second to fourth sessions included psychological education regarding OCD and case formulation, and the fifth to eighteenth sessions were devoted to treatment procedures tailored for each patient, such as exposure and response prevention (if applicable), and shaping method combined with the coordination of the patients’ circumstances including family relationships, working conditions, and school adjustments. These practical treatments could be shortened according to the patient’s conditions and improvement. The last two sessions included an introduction to relapse prevention. Therefore, the maximum number of sessions should be limited to 20. In this study, all OCD patients except for the dropout cases completed CBT, ranging from 11 to 20 sessions. All therapists who took part in this study completed the Improving Access to Psychological Therapies project at Chiba University (42). The quality of CBT was monitored through weekly supervisions by a psychiatrist.

### Assessment

#### Severity of Obsessive–Compulsive Symptoms

The severity of obsessive–compulsive symptoms was assessed using the Y-BOCS interview scale. Y-BOCS was rated by a psychiatrist (Akiko Nakagawa) during pretreatment and then rated by a therapist during and after treatment. Although we were not able to use the blind rating procedure, Akiko Nakagawa, an expert on this scale, trained and supervised the therapists’ ratings. Y-BOCS was assessed at pretreatment, mid-treatment (at sixth to eighth session), and posttreatment.

#### Autistic Traits

Autistic traits were assessed using AQ. AQ is a brief, self-rated scale measuring five domains of autistic traits covering social skills, attention switching, attention to detail, communication, and imagination. This scale was assessed at pretreatment.

#### General Mood State and Impairment

The severity of depression and anxiety were assessed using the Patient Health Questionnaire-9 (PHQ-9) (43) and the Generalized Anxiety Disorder-7 (GAD-7) (44), respectively. In addition, the severity of impairment due to OCD was assessed using the Sheehan Disability Scale (SDS) (45). These three scales were assessed at pretreatment, mid-treatment (at sixth to eighth session), and posttreatment.

#### Response and Remission Rate

In this study, a remission was defined as attaining a posttreatment Y-BOCS score of 12 and under (38). Those who remitted were assigned to the remission group, and those who did not remit were assigned to the non-remission group.

### MRI Data Acquisition

Thirty-one subjects [13 patients in the OCD (ASD+) group and 18 patients in the OCD (ASD−) group] underwent T1-weighted MRI with a scanner equipped with a 32-channel phased-array head coil (Discovery MR750 3.0T; GE Healthcare, Waukesha, WI, USA). Eight patients were excluded for contraindications to MRI (e.g., claustrophobia, medical implant or other non-removable metal inside the body, or decision not to participate in MRI scanning). MRI data were collected by 3D fast spoiled gradient-echo sequence using the following parameters: 3.164 ms echo time, 8.124 ms repetition time, 15° flip angle, 256 × 256 acquisition matrix, 1 mm slice thickness, 25.6 cm × 25.6 cm field of view, one excitation step, 31.25 kHz bandwidth, 420 ms inversion time, and an acceleration factor of 2. The data was preprocessed using the VBM8 toolbox,^1^ which is an extension of the unified segmentation model consisting of spatial normalization, bias field correction, and tissue segmentation (46). We processed the MRI data using Statistical Parametric Mapping 12 (SPM12, Wellcome Institute of Neurology, University College London, UK^2^) running under MATLAB R2015b (MathWorks Inc., Natick, MA USA). Registration to the stereotaxic space of the Montreal Neurological Institute (MNI) consisted of linear affine transformation and non-linear deformation using high-dimensional Diffeomorphic Anatomical Registration through Exponential Lie Algebra normalization (47). The normalized and segmented images were modulated by applying a non-linear deformation, which allows the comparison of absolute amounts of tissue corrected for individual differences in brain size (48). Finally, bias-corrected, modulated, and warped tissue maps were smoothed with an 8-mm full width at half maximum Gaussian kernel. The voxel resolution of smoothed images was 1.5 mm × 1.5 mm × 1.5 mm.

### Statistical Analysis

#### Effectiveness of CBT for OCD Patients with and without ASD

We performed statistical analysis using R 3.3.2 (R Core Team, 2016, Vienna, Austria). Our first purpose was to investigate whether comorbidity with ASD might significantly affect treatment outcome. For the primary analysis, we compared the two groups [OCD (ASD+) and OCD (ASD−) groups] using a multilevel linear mixed model for three time points (pre-, mid-, and posttreatment) of the Y-BOCS total score *via* the lme4 package in R (49). We constructed two-sided 95% confidence intervals to maintain a 5% significance level. The values at pre-, mid-, and posttreatment were the outcome measures, and time was included as fixed effects. We also included the effects of the interaction between groups and time in the model. We included gender, age, age at onset, the baseline values of the outcome measures, and PHQ-9 and GAD-7 in the model as covariates. Participants were treated as random effects (to allow for clustering of data within each participant). We estimated standardized effect sizes and corresponding 95% confidence intervals for each group, as well as standardized effect sizes between both groups at each time point (pre-, mid-, and posttreatment) from these models. For the secondary analysis, we repeated the primary analysis for the SDS total scores using the same method as outlined above. We then calculated the percentages of treatment remitters by comparing remission rates between OCD patients with and without ASD.

#### Pretreatment Brain Structural Abnormalities Using MRI Comparisons

We performed statistical analysis of the MRI data with SPM12, which implemented a general linear model. A two-sample *t*-test for non-paired samples was used to compare group differences [OCD (ASD+) and OCD (ASD−) groups; non-remission and remission] in whole brain volumes. Age, age at onset, gender, and pretreatment YBOCS score were input as covariates for comparing the OCD (ASD+) and OCD (ASD−) groups. In order to examine the potential neurostructural predictors of CBT outcome, we compared the structural differences between non-remission and remission groups. Therefore, age, age at onset, gender, pretreatment Y-BOCS score, as well as total AQ score were also input as covariates for comparing non-remission and remission groups, as autistic traits might affect brain alterations. Several studies reported that comorbid major depression and other anxiety disorders could contribute to brain alterations among OCD patients [e.g., Ref. (22, 50–52)]. Specifically, Cardoner et al. (51) reported that comorbidity with major depression in OCD patients showed decreased gray matter volume in the medial OFC and abnormal structural covariances with other limbic and paralimbic regions. Therefore, in the subsequent analysis, we input the scores of PHQ-9 and GAD-7 as nuisance covariates. The initial voxel threshold was set at 0.001, uncorrected for multiple comparisons. Clusters were considered as significant when falling below cluster-corrected *p* (family wise error: FWE) = 0.05. The anatomic location of each resulting cluster was determined using the MRI Atlas (53).

## Results

### Effectiveness of CBT for OCD Patients with and without ASD

Table 1 summarizes the detailed demographic data of patients involved in this study. The OCD (ASD+) group had many less females compared to the OCD (ASD−) group. As for the baseline clinical measures shown in Table 2, the OCD (ASD+) group had a significantly higher score on total AQ scores, social skills, and communication subscales of AQ compared to the OCD (ASD−) group.

**Table 1:** Demographic data for psychometric analysis.

	Obsessive–compulsive disorder (OCD) [autism spectrum disorder (ASD)+]	OCD (ASD−)	

	*n* **=** 15	*n* = 22	
**Mean (SD)**			

Age	29.53 (7.25)	34.09 (7.36)	*t* = 1.86, n.s.
Age at onset	19.07 (5.27)	25.00 (8.70)	*t* = 2.58*
Duration of OCD	10.47 (7.29)	9.14 (6.89)	*t* = 0.56, n.s.
Intelligence quotient	100.67 (10.38)	100.90 (12.09)	*t* = 0.06, n.s.
Number of sessions	16.87 (2.85)	16.05 (3.71)	*t* = 0.76, n.s.

**No. (%)**			

Gender^a^	4 (26.67)	17 (77.27)	*Z* = 3.05**
**Comorbidity**			
Major depressive disorder	5 (66.67)	1 (4.54)	*Z* = 2.33*
Social anxiety disorder	2 (13.33)	1 (4.54)	*Z* = 0.34, n.s.
Dysthymic disorder	1 (6.67)	–	–
Posttraumatic stress disorder	1 (6.67)	–	–
**Medication at pretreatment**			
Medication-free	4 (26.67)	2 (9.09)	*Z* = 1.42, n.s.
SSRI	9 (60.00)	16 (72.73)	*Z* = 0.81, n.s.
Antipsychotic augmentations	5 (33.33)	5 (22.73)	*Z* = 0.71, n.s.
Major tranquilizers	5 (33.33)	8 (36.36)	*Z* = 0.19, n.s.
Clomipramine	3 (20.00)	2 (9.09)	*Z* = 0.95, n.s.

**Mean (SD)**			

CP equivalent doses^b^	110.58 (82.03)	104.88 (55.25)	*t* = 0.15, n.s.

*^a^Number of women*.

*^b^Mean of chlorpromazine equivalent doses of major tranquilizers for each patient*.

*p < 0.05.

**p < 0.01.

**Table 2:** Baseline clinical measures of outcome scores for psychometric analysis.

		Obsessive–compulsive disorder (OCD) [autism spectrum disorder (ASD)+] *n* = 15	OCD (ASD−) *n* = 22	
Y-BOCS	Total	26.00 (2.65)	25.86 (3.96)	*t* = 0.12, n.s.
Y-BOCS	Obsession	13.40 (1.80)	13.27 (2.33)	*t* = 0.18, n.s.
Y-BOCS	Compulsion	12.60 (1.24)	12.59 (1.92)	*t* = 0.02, n.s.

AQ	Total	29.73 (5.69)	22.65 (7.26)	*t* = 3.12**
	Social skills	6.27 (2.12)	4.60 (2.46)	*t* = 2.10*
	Attention switching	6.93 (1.58)	6.30 (1.92)	*t* = 1.04, n.s.
	Attention to details	5.47 (1.76)	5.40 (1.64)	*t* = 0.12, n.s.
	Communication	6.00 (1.93)	2.80 (2.33)	*t* = 4.32**
	Imagination	5.07 (2.28)	3.55 (2.33)	*t* = 1.92, n.s.

PHQ-9		13.20 (4.51)	9.86 (6.69)	*t* = 1.68, n.s.
GAD-7		12.67 (3.66)	10.27 (4.90)	*t* = 1.61, n.s.
SDS		6.49 (1.88)	6.52 (1.82)	*t* = 0.06, n.s.

*Values enclosed in parentheses represent SDs*.

*p < 0.05.

**p < 0.01.

*Y-BOCS, Yale–Brown Obsessive–Compulsive Scale; AQ, autism-spectrum quotient; PHQ-9, Patient Health Questionnaire-9; GAD-7, generalized anxiety disorder-7; SDS, Sheehan Disability Scale*.

**Figure 2:**
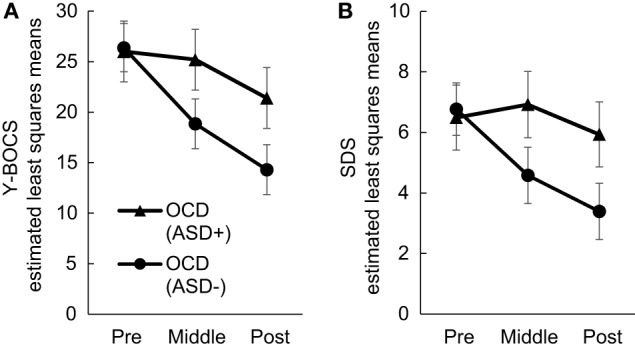
Changes in Yale–Brown Obsessive–Compulsive Scale **(A)** and Sheehan Disability Scale **(B)** scores in each group during Cognitive Behavioral Therapy.

Our first purpose was to investigate whether comorbidity with ASD may significantly affect treatment outcome. We found significant interactions between time and group in the total Y-BOCS and SDS mean scores (Y-BOCS, *p* < 0.001; SDS, *p* < 0.001). There was a statistically significant difference in the total Y-BOCS and SDS mean scores at mid- and posttreatment between the OCD (ASD+) and OCD (ASD−) groups (Table 3). Figures 2A,B plot the estimated means of the Y-BOCS and SDS scores at each time point. We initially focused on the Y-BOCS total score: the effect of group differed significantly at mid-treatment and posttreatment. CBT for the OCD (ASD−) group was already effective at mid-treatment, but not for the OCD (ASD+) group (Figure 2A). The results of the SDS scores were more striking. Over the course of the CBT sessions, the OCD (ASD+) group felt a greater sense of disability (Figure 2B). Therefore, the comorbidity with ASD seems to affect the CBT outcome in terms of severity of obsessive–compulsive symptoms and impairment in daily life due to OCD. In addition, OCD patients with ASD had a significantly lower remission rates compared to OCD patients without ASD (13.33 vs. 54.54%).

**Table 3:** Linear mixed model for Y-BOCS and SDS.

	Obsessive–compulsive disorder (OCD) [autism spectrum disorder (ASD)+]		OCD (ASD−)		Mean group difference	Standardized effect size		
	M	SD	M	SD		Hedges’ g	Lower 95% CI	Upper 95% CI
**Y-BOCS**								
Pre	25.41	5.69	26.49	5.56	−1.07	−0.19	−0.84	0.47
Middle	24.61	5.69	19.13	5.74	5.48	0.94	0.25	1.63
Post	20.81	5.69	14.65	5.80	6.16	1.05	0.35	1.74
**SDS**								
Pre	6.33	1.91	6.69	1.91	−0.36	−0.18	−0.84	0.47
Middle	6.80	1.95	4.64	2.07	2.16	1.04	0.35	1.74
Post	5.78	1.91	3.45	2.07	2.33	1.14	0.43	1.84

*M, estimated mean; SD, estimated SD; CI, confidence interval; Y-BOCS, Yale–Brown Obsessive–Compulsive Scale; SDS, Sheehan Disability Scale*.

*Gender, age, age at onset, baseline values of outcome measures, and Patient Health Questionnaire-9 and Generalized Anxiety Disorder-7 were included in the model as covariates. Effect sizes retained + and − signs to indicate the direction of the OCD (ASD+)—OCD (ASD−) differences*.

### Pretreatment MRI Comparison: OCD Patients with and without ASD, and Remitted OCD Patients and Non-Remitted OCD Patients

**Figure 3:**
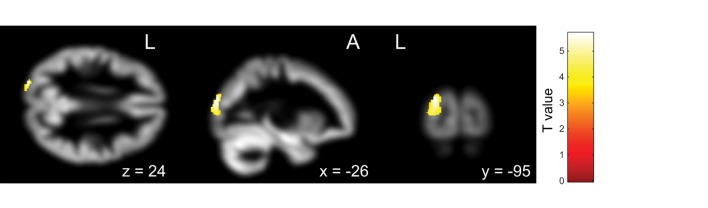
Pretreatment differential gray matter volumes between obsessive–compulsive disorder (OCD) patients with autism spectrum disorder (ASD) (*n* = 13) and without ASD (*n* = 18). Brain regions containing loci of decreased gray matter volume for OCD patients with ASD relative to OCD patients without ASD. Age, age at onset, gender, and pretreatment Yale–Brown Obsessive–Compulsive Scale implemented as covariates. *p* < 0.05, corrected for multiple comparisons, family wise error, and *k* (voxels in cluster) >647 voxels.

**Figure 4:**
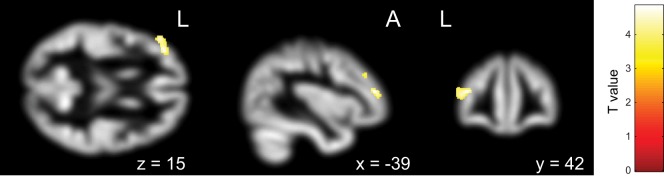
Pretreatment differential gray matter volumes between the non-remission group (*n* = 14) and the remission group (*n* = 17). Brain regions containing loci of decreased gray matter volume in the non-remission group relative to the remission group. Age, age at onset, gender, pretreatment Yale–Brown Obsessive–Compulsive Scale, and Autism-Spectrum Quotient implemented as covariates. *p* < 0.05, corrected for multiple comparisons, family wise error, and *k* (voxels in cluster) >759 voxels.

First, the OCD (ASD+) group had significantly less gray matter volume in the left occipital lobe than the OCD (ASD−) group (Table 4; Figure 3). Second, the non-remission group exhibited a significantly smaller volume of gray matter in the left middle frontal gyrus (Brodmann area 10, 46) than the remission group (Table 5; Figure 4). In additional analysis with PHQ-9 and GAD-7 as extra covariates, the finding of the comparison between the OCD (ASD+) and OCD (ASD−) groups did not survive strict correction for multiple comparisons. In a second analysis with PHQ-9 and GAD-7 as extra covariates, the finding of the comparison between the non-remission and remission groups survived strict correction for multiple comparisons (Table S1 and Figure S1 in Supplementary Material).

**Table 4:** Differences in gray matter volumes between obsessive–compulsive disorder (OCD) patients with and without autism spectrum disorder (ASD).

Brain region^a^	Brodmann area	Direction of correlation	Montreal neurological institute coordinates			*Z-*value	Voxels in cluster	*p* (FWE)^b^
			*x*	*y*	*z*			
**OCD (ASD+) (*n* = 13) vs. OCD (ASD−) (*n* = 18)**								
L. superior occipital gyrus	19	Decreased in OCD (ASD+)	−26	−94	24	4.51	647	0.031
L. middle occipital gyrus	19		−29	−96	10	4.06		
L. superior occipital gyrus	18		−15	−102	15	3.17		

*OCD (ASD+) = OCD patients with ASD, OCD (ASD−) = OCD patients without ASD; L, left*.

*^a^A voxel-based whole-brain analysis*.

*^b^Family wise error (FWE) correction for multiple comparison*.

**Table 5:** Differences in gray matter volumes between non-remitted obsessive–compulsive disorder (OCD) patients and remitted OCD patients.

Brain region^a^	Brodmann area	Direction of difference	Montreal neurological institute coordinates			*Z*-value	Voxels in cluster	*p* (FWE)^b^
			*x*	*y*	*z*			
**OCD (non-remitted) (*n* = 17) vs. OCD (remitted) (*n* = 14)**								
L. middle frontal gyrus	10	Decreased in OCD (non-remitted)	−39	42	15	4.00	759	0.016
L. middle frontal gyrus	46		−50	39	16	3.77		
L. middle frontal gyrus	10		−32	51	6	3.74		

*OCD (non-remitted) = OCD patients who did not reach remittion after CBT, OCD (remitted) = OCD patients who remitted after cognitive behavioral therapy (CBT); L, left*.

*^a^A voxel-based whole-brain analysis*.

*^b^Family wise error (FWE) correction for multiple comparison*.

## Discussion

### Effectiveness of CBT for OCD Patients with and without ASD

Our first goal was to examine the effectiveness of CBT for OCD patients with and without ASD, one of the risk factors for the resistance to CBT. The OCD patients with ASD responded significantly less well to CBT. We also observed significantly lower remission rates. As expected, these results were consistent with previous studies (6, 7). In addition, we found that OCD patients with ASD felt a greater sense of disability compared with OCD patients without ASD over the course of the CBT sessions. These results indicated that OCD patients with ASD could not get sufficient benefit from the treatment, leading them to feel a sense of disability and daily impairment. Another interpretation of this result was that their sensitivity to self-rated disability and daily impairment might negatively affect their CBT outcome. Therefore, a greater sense of disability over the course of CBT might be a factor contributing to treatment resistance to CBT for OCD patients with ASD.

However, these findings should not be over-interpreted as being due to comorbid major depression or other anxiety disorders. Although we partialed out the PHQ-9 and GAD-7 scores, and we found significant differences of the Y-BOCS and SDS scores between the two groups, we did not focus on group comparison without depression and other anxiety disorders. Previous studies showed that more distribution of comorbid major depression or other anxiety disorders were natural for the OCD patients with ASD [e.g., Ref. (6)]. Therefore, we thought that the result of this study showing more distribution of comorbid major depression or other anxiety disorders was not surprising and was natural for the OCD (ASD+) group. In addition, a previous meta-analysis, which investigated the predictors of CBT outcome for OCD, reported that, as symptom-specific variables, age at onset, and duration of illness were inconsistent, and more consistent findings were greater symptom severity and hoarding subtype serving as predictors of poorer treatment response (54). With regard to comorbidity, the presence of a personality disorder was found to consistently predict poorer treatment response, rather than comorbid depression and tics (54). Although we did not investigate other potential predictors mentioned by Keeley et al. (54), and thus cannot conclude that autistic traits directly affect CBT outcome, comorbidity with ASD may have an impact on CBT outcome.

### Common Neural Abnormalities of ASD Traits among OCD Patients

Our second goal was to investigate the structural abnormalities profile of the pretreatment brain associated with the CBT outcomes. In the present study, we found that OCD patients with ASD had a significantly smaller gray matter volume than OCD without ASD in the left occipital lobe, which plays an important role in visuospatial processing. In a previous meta-analysis of structural MRI using an age- and gender-matched subgroup, the most prominent disorder-specific finding was that the right putamen and insula were increased in gray matter volume of OCD patients but decreased in ASD patients, relative to control subjects (55). Another finding was that the left superior frontal gyrus was smaller in gray matter volume of OCD patients than in ASD patients (55). In our study, however, we did not observe such volume changes in the frontal lobe nor in the basal ganglia between OCD patients with and without ASD. This may be attributed to the comorbidity between OCD and ASD. In our study, we compared OCD patients with and without ASD to elucidate how autistic traits influence the CBT outcomes. Our results revealed that comorbid patients with both OCD and ASD had less gray matter volume in the left occipital lobe compared to OCD patients without ASD.

Menzies et al. (11) showed that there are also other consistent abnormalities in the middle occipital cortices, parietal cortices, and cerebellum aside from the “affective” orbitofronto-striatal circuit. This suggests that OCD involves more distributed large-scale brain systems. Several studies have suggested that parieto-occipital abnormalities in patients with OCD might be associated with obsessive–compulsive symptoms (13, 19, 26, 56). In a functional MRI study, Nakao et al. (57) also observed increased activation in both the occipital and parietal lobes after treatment. However, these studies did not consider the effect of autistic traits. Our results indicate that autistic traits in OCD patients might affect the parieto-occipital abnormalities. However, when considering the result after having partialed out the severity of depression and anxiety, we could not detect the findings of smaller gray matter volume in the left occipital lobe in the OCD (ASD+) group. Therefore, the decrease in gray matter volume in the occipital lobe might be affected by depression and anxiety. Comorbidity with ASD may affect the perceived internal state of mind (i.e., depression and anxiety), or the ability of emotion regulation, although we did not find significant differences in the severity of depression and anxiety between the two groups [OCD (ASD+) vs. OCD (ASD−)]. In other words, the severity of depression and anxiety [i.e., the ability to regulate emotion, which might be impaired in OCD patients with ASD (58)] might be associated with the decreased gray matter volume in the left occipital lobe.

### Pretreatment Brain Characteristics in OCD Patients Who Did Not Remit after CBT

On the other hand, when we focused on the differences between the non-remission and remission groups while taking autistic traits into considerations, we observed that, at pretreatment, the non-remission group displayed a smaller gray matter volume than the remission group in the left middle frontal gyrus (Brodmann area 10, 46). These findings survived after having partialed out the severity of depression and anxiety. Anatomically speaking, these areas correspond to DLPFC, which has an important role in executive functions. These regions are involved in goal representation and attention selection, as well as in response inhibition and the maintenance of stimulus representations in the presence of distracting or interfering events (59). Traditionally, DLPFC was considered to be responsible for planning functions, and together with parietal regions, has been considered to be a part of the dorsolateral prefronto-striatal “executive” circuit (9, 11, 13). A revised model for OCD proposed by Menzies et al. (11) showed that the brain pathology of OCD is not limited to the orbitofronto-striatal “affective” circuit associated with limbic structures (such as the amygdala), but also involves abnormalities including DLPFC that may represent the dorsolateral prefronto-striatal “ executive” circuit.

In this analysis, we did not observe any differences in the limbic system between the non-remission and remission groups. However, we did find a smaller gray matter volume in the left DLPFC of the non-remission group. These results indicate that the abnormalities in DLPFC negatively affect the CBT outcome regardless of the severity of autistic traits. Indeed, OCD patients present impairments in several cognitive domains (i.e., memory, attention, flexibility, inhibition, verbal fluency, planning, and decision-making) (60). Planning and non-verbal memory are mildly impaired with effect sizes ranging from −0.44 to −0.73 (61–63). When we focus on the process of CBT, at first, unreasonable obsession and excessive compulsion may, for OCD patients with ASD, seem completely reasonable in light of their own judgment. However, when some of them experience therapeutic alliance, and their learning process accelerates over time, they may come to question these obsessions or compulsions. However, if the abovementioned cognitive domains are impaired, this improvement in cognitive inflexibility may not occur easily. Cognitive inflexibility in OCD patients can arise from a firm conviction about their irrational cognitions, which prevent them from collecting new information and changing these firm convictions into normal cognitive evaluations. Difficulties in encoding and transforming an experience into memory might disturb the updating process for irrational cognitions. As a result, cognitive inflexibility can persist or even worsen.

Moreover, the OCD patients with ASD (who did not benefit from CBT) perceived greater disability as measured by SDS. As Bejerot et al. (8) noted, cognitive inflexibility is one of the autistic traits in OCD patients, and this may affect the CBT outcome. Although we did not explore the relationship between the severity of autistic traits and cognitive deficits, both together might have determined the resistance to CBT. Thus, there might be these unexplored factors associated with CBT outcome in this study (e.g., the relationship between autistic traits and cognitive deficits); however, it is possible that DLPFC (where we found smaller gray matter volume in the non-remission group) may be associated with the CBT outcome. The underlying factors of ASD are considered to be deficits in theory of mind, weak central coherence, and executive dysfunction (64). Previous studies have shown that these deficits may partially explain variations in the course of social development, communication difficulties, restricted interests, and repetitive behaviors in ASD (65). Therefore, the cognitive deficits in OCD and ASD might partially overlap, and thus, when autistic traits were added to OCD, these cognitive deficits might worsen, which could result in a poor CBT outcome. We thought that further studies investigating the overlapping and differences of these cognitive deficits between OCD and ASD might significantly improve our understanding of the mechanisms of both OCD and ASD.

### Limitations and Conclusion

The results of this study must be interpreted with caution due to several limitations. First, the modest sample size may have limited our ability to detect differences in other regions of the brain. This modest sample size was largely due to our decision to focus on the differences between OCD patients with and without ASD. Second, in the present study, the neuropsychological mechanism and neuroplasticity (changes in gray matter volume induced by CBT) remain unclear because we did not acquire neuroimaging data after CBT treatment. Therefore, we need to determine the causality between brain structure and clinical features of OCD through longitudinal neuroimaging studies. This will allow us to examine the effects of CBT based on the changes in morphology, functioning, and connectivity using not only structural MRI, but also functional MRI. Third, we did not control the medication status of our OCD patients, which may have added variability to our data. In addition, comorbidity with mood disorders and anxiety disorders in OCD could negatively affect the treatment outcome (66). In our study, we did not control the comorbid conditions shown in Table 1 because we adopted broad inclusion criteria to build a clinical setting and because we could not find any significant differences in the severity of depression and anxiety through the process of the group comparison analysis. However, the distribution of those who presented comorbidities between the OCD (ASD+) and OCD (ASD−) groups was differed significantly, which may have negatively affected the CBT outcome. Future studies are needed, controlling the medication status and comorbid conditions, to clarify both short- and long-term predictors of treatment outcome. Fourth, our study plan did not include a healthy control group or a group with patients only suffering from ASD. In order to interpret our results more clearly, research comparing OCD groups, ASD groups, healthy control groups, and placebo-treated groups are needed in the future. Fifth, we focused on comorbidity with high-function ASD, because one of our inclusion criteria was a total IQ over 80. Another limitation of the present study was the lack of blind independent ratings of Y-BOCS. Furthermore, we did not assess homework compliance, which might be associated with motivation required by CBT. Moreover, in the current study, we did not analyze neuropsychological abilities such as attention control, attention switching, planning, and so on. Further studies are needed to clarify the relationships between the smaller gray matter volume in DLPFC and its function in the non-remission group.

Despite these limitations, this study is the first to examine the neurostructural predictors of CBT for OCD, while taking autistic traits into account. In conclusion, group comparison analyses between OCD (ASD+) and OCD (ASD−) revealed a smaller gray matter volume in the left occipital lobe of the OCD (ASD+) group. In addition, group comparison analyses, after having partialed out autistic traits, between the non-remission and remission groups revealed a smaller gray matter volume in the left DLPFC of the non-remission group. These results indicate that CBT outcomes for OCD patients may not only be attributable to autistic traits but also to “executive” circuits. We conclude that distributed large-scale brain regions represented by the dorsolateral “executive” circuit may explain neurobiological mechanisms of resistance to CBT outcome. Autistic traits might affect cognitive deficits, which are associated with the “executive” circuits. Therefore, investigating the overlapping and differences of these cognitive deficits between OCD and ASD might shed some light on the pathophysiology of these disabling disorders, and this would also be promising for an improvement in CBT outcome.

## Informed Consent

All procedures followed were in accordance with the ethical standards of the responsible committees on human experimentation (institutional and national) and with the Helsinki Declaration of 2013, and the appropriate revisions at the time of the investigation. Informed consent was obtained from all patients included in the study.

## Ethics Statement

The Institutional Research and Ethics Committee of the Graduate School of Medicine, Chiba University, approved the study. We obtained written and informed consent from each subject regarding the study before the assessments began.

## Author Contributions

Study concept and design: AT and AN. Acquisition of data: YH, KA, FO, SN, YT, KM, and YM. Statistical analysis and interpretation of data: AT, YH, and YT. Drafting of the manuscript: AT. Critical revision of the manuscript: AT, YH, and AN. Study supervision: YH, KA, FO, SN, KM, YM, MI, ES, and AN. All authors read and approved the version to be published.

## Conflict of Interest Statement

The authors declare that the research was conducted in the absence of any commercial or financial relationships that could be construed as a potential conflict of interest.

## Footnotes

^1^http://dbm.neuro.uni-jena.de/vbm/.

^2^http://www.fil.ion.ucl.ac.uk/spm/software/spm12/.

## Supplementary Material

Figure S1 Pretreatment differential gray matter volumes between the non-remission group (*n* = 14) and the remission group (*n* = 17). Brain regions containing loci of decreased gray matter volume in the non-remission group relative to the remission group. Age, age at onset, gender, pretreatment Yale–Brown Obsessive–Compulsive Scale, Autism-Spectrum Quotient, Patient Health Questionnaire-9, and Generalized Anxiety Disorder-7 implemented as covariates. *p* < 0.05, corrected for multiple comparisons, family wise error, and *k* (voxels in cluster) >565 voxels.

## Abbreviations

ACC, anterior cingulate cortex; AQ, autism-spectrum quotient; ASD, autism spectrum disorder; CBT, cognitive behavioral therapy; DLPFC, dorsolateral prefrontal cortex; FWE, family wise error; GAD-7, generalized anxiety disorder-7; IQ, intelligence quotient; MRI, magnetic resonance imaging; OCD, obsessive–compulsive disorder; OFC, orbitofrontal cortex; PHQ-9, patient health questionnaire-9; ROI, region of interest; SCID-I, structured clinical interview for DSM-IV axis I disorders; SDS, Sheehan Disability Scale; WAIS-III, Wechsler adult intelligence scale-III; Y-BOCS, Yale–brown obsessive–compulsive scale.
